# The Consequences of a Lab Escape of a Potential Pandemic Pathogen

**DOI:** 10.3389/fpubh.2014.00116

**Published:** 2014-08-11

**Authors:** Lynn C. Klotz, Edward J. Sylvester

**Affiliations:** ^1^The Center for Arms Control and Non-Proliferation, Washington, DC, USA; ^2^Science and Medical Journalism, Walter Cronkite School of Journalism and Mass Communication, Arizona State University, Phoenix, AZ, USA

**Keywords:** potential pandemic pathogen, lab-acquired infection, H5N1, H7N9, gain of function

In letters to the journals Science and Nature (1, 2), 22 virologists notified the research community of their interest in expanding research to develop strains of the already deadly H7N9 Asian influenza virus that would be transmissible via aerosols among mammals, thus creating potential pandemic pathogens. PPPs are defined as pathogens that are potentially highly contagious, potentially highly deadly, and not currently present in the human population. Mammalian contagious avian flu, the 1918 pandemic flu, and SARS are examples. The letter writers cite their scientific reasons for the need for such research, much the same reasons as given by those working on similar projects for the H5N1 avian flu virus (3, 4). This new proposed research signals wider interest in making dangerous influenza viruses (5, 6) contagious in mammals via respiratory aerosols. At present, there are no international regulations or guidelines in place to decide whether such a research project should proceed.

Now is the time to address the next critical question: what is the likelihood that one of these viruses will escape from a lab and seed the very pandemic the researchers claim they are trying to prevent? As we shall estimate, that probability could be as high as 27%, a risk too dangerous to live with.

First, from the calculations in two in-depth pandemic risk analyses (7–9), there is a substantial probability that a pandemic with over a 100-million fatalities could be seeded from an undetected lab-acquired infection (LAI), if a single infected lab worker spreads infection as he moves about in the community. From the Klotz (2014) analysis, there is about a 1–30% probability, depending on assumptions, that, once infected, the lab worker will seed a pandemic. This large probability spread arises from varying the average number of people infected by an infected person between 1.4 and 3.0 (*R*_0_, in standard epidemiology notation), varying the details of commutes to and from work on public transportation, and whether infected acquaintances are quarantined before spreading infection. The Merler (2013) study, based on a computer-generated population grid of size and varying density of the Netherlands, supports our concern over a lab escape not being detected until it is too late: “there is a non-negligible probability (5–15%), strongly dependent on reproduction number and probability of developing clinical symptoms, that the escape event is not detected at all.”

Different methodologies were used in the Klotz (2014) and Merler (2013) risk analyses. Additional analyses are needed using other methodologies, such as the mathematical model employed for SARS (10), which hopefully will lead to some consensus on risk. The Klotz and Merler studies, however, are the first to raise these concerns and point to valid issues about the potential risks from a single LAI.

Given such a dire predicted outcome by the existing studies, the critical question is: what is the probability that a worker acquires an undetected infection in the lab in the first place? To answer this question, we reproduce here one part of the Klotz (2014) analysis: the probability of an escape through an LAI from at least one of the many labs expected to be involved in this research enterprise.

A 2013 Centers for Disease Control report is a significant source of recent data on LAIs (11). The report documents four undetected or unreported LAIs in registered US Select Agent, high-containment BSL-3 labs between 2004 and 2010. An undetected or unreported LAI implies an escape when the infected person leaves the lab. The report identifies an average of 292 registered Select Agent BSL-2, BSL-3, and BSL-4 labs operating over those 7 years, for a total of 292 × 7 = 2,044 lab years. Unfortunately, the study does not break down numbers into BSL-2, BSL-3, and BSL-4 labs or lab years.

Thus, the probability of escape for a single year, *p*_1_, can only be calculated as 4 LAIs/2,044 lab years = 0.002 or 0.2% per lab per year. This is clearly an underestimate since BSL-2 and BSL-4 labs contribute to the denominator. (The denominator used here, 2,004, equals the number of BSL-2 plus number of BSL-3 plus number of BSL-4 labs. But the denominator in our calculation should be just the number of BSL-3 labs, so the denominator is overestimated and the percent escape is then underestimated. Although requested, the CDC has not supplied us with the number of BSL-3 labs for us to do the exact calculation.) This basic probability is consistent with that for SARS escapes in Asia through LAIs (12) and with all known escapes from BSL-4 labs in the Soviet Union from LAIs and Great Britain from a mechanical failure (13).

To illustrate potential risk, the probability of no escape from a single lab in a single year is (1 − *p*_1_), so

(1) $$p_{\mathrm{no}}={\left(1-\;p_{1}\right)}^{N\;\times \;Y}$$

is the probability of no escape from *N* labs in *Y* years. And

(2) $$p_{\text{at}\;\text{least}\;\text{one}}=\;1-{\left(1-\;p_{1}\right)}^{N\times Y}$$

is the probability of at least one escape from *N* labs in *Y* years.

Given the Science and Nature articles listed above (1, 2), it is reasonable to assume that at least 10 labs will undertake this research and that this work would continue for 10 years, so

(3) $$p_{\text{at}\;\text{least}\;\text{one}}=\;1-{\left(1-0.002\right)}^{10\times 10}=\;0.18$$

or an 18% likelihood of at least one escape from at least one lab for the whole research enterprise, almost 100-times greater than the likelihood for a single lab in a single year.

We noted above that the probability *p*_1_ = 0.2% is conservative, estimated from the CDC data alone. The first Department of Homeland Security risk assessment for the planned National Bio- and Agro-Defense Facility in Manhattan, Kansas estimated a significantly higher escape risk, over 70% likelihood for the 50-year life of the facility (14), which works out to be a basic probability of escape, *p*_1_ = 2.4% per year. The National Research Council (14) overseeing the risk assessment remarked “The … estimates indicate that the probability of an infection resulting from a laboratory release of FMDv from the NBAF in Manhattan, Kansas approaches 70% over 50 years (see Figure 3-1) with an economic impact of $9–50 billion. The committee finds that the risks and costs could well be significantly higher than that…” While the DHS subsequently lowered the escape risk to 0.11% for the 50-year lifetime (14), the NRC committee (14) was highly critical of the new calculations: “The committee finds that the extremely low probabilities of release are based on overly optimistic and unsupported estimates of human error rates, underestimates of infectious material available for release, and inappropriate treatment of dependencies, uncertainties, and sensitivities in calculating release probabilities.” We have more trust in the NRC committee conclusions, as they have no skin in the game.

With this higher number, which we take as a worst-case scenario, the likelihood of at least one escape from 10 labs in 10 years becomes 91%, almost a certainty. It follows that, if the likelihood of one LAI leading to a pandemic is 30% in the worst-case scenario, the likelihood of an LAI-caused pandemic resulting from this whole research enterprise could be as high as 30 × 91% = 27%, a likelihood that is too dangerous to live with, as we noted. While this represents a worst-case scenario, it is not improbable.

Recent self-reported mistakes at the CDC (15), involving a particularly deadly strain of anthrax removed from BSL-3 containment and H5N1 Asian bird flu released from the CDC laboratories altogether, lend support to our concern that the probability of escape may be much greater than the 0.2% per lab per year from just LAIs. The CDC report spawned a congressional inquiry (16) and led to dozens of newspaper articles with concerns about lack of safety in high-containment laboratories.

Our concern is shared by many virologists and epidemiologists. A recent letter to the President of the European Commission (17) co-signed by 56 scientists from more than a dozen countries warned, “The probabilities of a lab accident that leads to a global spread of an escaped mutated virus are small but finite, while the impact of global spread could be catastrophic.” The European Centre for Disease Prevention and Control (18) weighed-in with its concerns as well, as did the Cambridge Working Group (19). It must be noted that some of the signers of the European Commission letter and the Cambridge Working Group’s consensus statement are the same.

The risk of a man-made pandemic from a lab escape is not hypothetical. Lab escapes of high-consequence pathogens resulting in transmission beyond lab personnel have occurred (20, 21). The historical record reveals lab-originated outbreaks and deaths due to the causative agents of the 1977 pandemic flu, smallpox escapes in Great Britain, Venezuelan equine encephalitis in 1995, SARS outbreaks after the SARS epidemic, and foot and mouth disease in the UK in 2007. Ironically, these labs were working with pathogens to prevent the very outbreaks that they ultimately caused.

Do benefits outweigh risks? Those who support PPP experiments either believe the probability of PPP escape is infinitesimal or the benefits in preventing a pandemic are great enough to justify the risk. In making decisions for what lines of research will lead to new knowledge, experts must rely on intuition honed by years of research in a particular field. In the case of this PPP research, in our opinion it would take extraordinary benefits and significant reduction of risk via extraordinary biosafety measures to correct such a massive overbalance of highly uncertain benefits to too-likely risks (Wain-Hobson, 2013).

Whatever number we are gambling with, it is clearly far too high a risk to human lives. This Asian bird flu virus research to develop strains transmissible via aerosols among mammals, and perhaps some other PPP research as well, should for the present be banned. We must emphasize that we have been considering only a very small subset of pathogen research. Most pathogen research should proceed unimpeded by unnecessary regulations.

Special precautions in BSL-4 laboratories for work with PPPs should be adopted (22). These would include:

- Training a full-time technical staff for work with PPPs. Experiments could be directed by scientists outside the laboratory using modern audio-video technology.
- Requiring the staff to follow up extended work shifts with periods of quarantine before they leave the containment area to assure that no PPP escapes from the containment area through an LAI.
- Restricting these PPP laboratories to remote locations, where an aerosol escape or other containment failure would pose the least risk of infecting an outside community.

We label BSL-4 laboratories with the special precautions, BSL-4+. While PPP experiments would be carried out primarily under BSL-4+ containment, BSL-3 containment with the special precautions might suffice for some work.

Given the global threat, the international community should insist on discussions leading to an international agreement that would require the strictest oversight to conduct this particular research anywhere. To place responsibility with the international community where it belongs and to provide maximum transparency, policy makers should require that international inspectors have access to facilities at any time on short notice.

As it stands, there is no proactive oversight nor regulations for this PPP research, so any and all of the world’s nations can carry out this dangerous work without regard to consequences. But consequences would be shared by all of us. In the meantime, insurance companies who routinely provide insurance for biological research should consider excluding such risky research from coverage.

## Conflict of Interest Statement

The authors declare that the research was conducted in the absence of any commercial or financial relationships that could be construed as a potential conflict of interest.
